# Low cerebrospinal fluid Amyloid-βeta 1–42 in patients with tuberculous meningitis

**DOI:** 10.1186/s12883-021-02468-2

**Published:** 2021-11-16

**Authors:** Giacomo Stroffolini, Giulia Guastamacchia, Sabrina Audagnotto, Cristiana Atzori, Mattia Trunfio, Marco Nigra, Alessandro Di Stefano, Giovanni Di Perri, Andrea Calcagno

**Affiliations:** 1 Amedeo di Savoia Hospital, Infectious Diseases Unit, Department of Medical Sciences, University of Turin, Turin, Italy; 2 Maria Vittoria Hospital, Unit of Neurology, Asl Città di Torino, Italy; 3 San Giovanni Bosco Hospital, Laboratory, Asl Città di Torino, Italy

**Keywords:** Tuberculosis, Meningitis, Alzheimer’s disease, Amyloid-beta, Neuromarkers, Dementia

## Abstract

**Background:**

Tuberculous meningitis (TBM) is an important disease leading to morbidity, disability and mortality that primarily affects children and immune-depressed patients. Specific neuromarkers predicting outcomes, severity and inflammatory response are still lacking. In recent years an increasing number of evidences show a possible role for infective agents in developing neurodegenerative diseases.

**Methods:**

We retrospectively included 13 HIV-negative patients presenting with TBM and we compared them with two control groups: one of patients with a confirmed diagnosis of AD, and one of those with syphilis where lumbar punctures excluded central nervous system involvement. Lumbar punctures were performed for clinical reasons and CSF biomarkers were routinely available: we analyzed blood brain barrier permeability (CSF to serum albumin ratio, “CSAR”), intrathecal IgG synthesis, (CSF to serum IgG ratio), inflammation (neopterin), amyloid deposition (Aβ1–42), neuronal damage (T-tau, P-tau, 14.3.3) and astrocytosis (S-100 β).

**Results:**

TBM patients were 83 % male and 67 % Caucasian with a median age of 51 years (24.5–63.5 IQR). Apart from altered CSAR (median value 18.4, 17.1–30.9 IQR), neopterin (14.3 ng/ml, 9.7–18.8) and IgG ratios (15.4, 7.9–24.9), patients showed very low levels of Aβ1–42 in their CSF (348.5 pg/mL,125-532.2), even lower compared to AD and controls [603 pg/mL (IQR 528–797) and 978 (IQR 789–1178)]. Protein 14.3.3 tested altered in 38.5 % cases. T-tau, P-tau and S100Beta were in the range of normality. Altered low level of Aβ1–42 correlated over time with classical TBM findings and altered neuromarkers.

**Conclusions:**

CSF Biomarkers from patients with TBM were compatible with inflammation, blood brain barrier damage and impairment in amyloid-beta metabolism. Amyloid-beta could be tested as a prognostic markers, backing the routine use of available neuromarkers. To our knowledge this is the first case showing such low levels of Aβ1–42 in TBM; its accumulation, drove by neuroinflammation related to infections, can be central in understanding neurodegenerative diseases.

**Supplementary Information:**

The online version contains supplementary material available at 10.1186/s12883-021-02468-2.

## Background

Central nervous system (CNS) infections are uncommon diseases characterized by significant morbidity, disability and mortality. Tuberculous meningitits (TBM) is the most severe manifestation of extrapulmonary infection by *Mycobacterium tuberculosis*. It is characterized by a slowly progressing granulomatous inflammation of the basal meninges, an inflammatory reaction that can lead to complications such as hydrocephalus, cerebral vascular infarction, cranial nerve palsy and, if untreated, death. Vulnerable populations are at higher risk of infection and complications. Rapid diagnosis and initiation of treatment is therefore necessary to reduce the high mortality and severe sequelae associated with the disease. Diagnosing TBM can be difficult as the symptoms are non-specific and they mimic other infections or vascular disorders. The identification of specific plasma or cerebrospinal fluid (CSF) biomarkers may be relevant for an early diagnosis and for prognosis. TBM is traditionally characterized by CSF pleocytosis, increased proteins, decreased glucose concentration. Although other CSF biomarkers have been investigated, none has reached clinical practice. S100b, NSE (neuron-specific enolase) and interleukins have been advocated to be predictive of disease’s severity and outcome [1–5]. Recently, several infectious agents have been called out to be possible triggers in causing neurodegenerative diseases, especially AD. The total burden of infectious agents has been linked to the development of AD in sporadic cases [6, 7]; this appears to be substantially due to microglia activation [8], long acting inflammation neuronal alteration, oxidative stress and amyloid-beta accumulation but also to a direct effect by infectious agents. Specifically, viruses from the *Herpesviridae* family have since long been called out to play a decisive role (together with APOE phenotype) [7, 9–11] in affecting disease onset and clinical progression. Other bacteria have also been suggested to have a causative role, including *Spirochetaceae*, *Chlamydia* and gram-negative bacteria [12, 13]. Recent reports suggest also a role for parasites in stimulating different pattern of inflammation [14] and no data have been outlined for fungi. Beside this, amyloid-beta has also been identified as a protein acting as an anti-infective peptide playing a direct role in the clearance of different infections in various animal models [15]. HSV6 appears to be capable of directly enhance the seeding and acceleration of amyloid-beta deposition despite a debated pathogenic potential [16]. Following important reviews [12, 17, 18], this suggestive hypothesis could link the accumulation of amyloid-beta during infection and the subsequent development of neurodegenerative disease. Aim of this analysis was to study the CSF concentrations of several biomarkers in patients with TBM.

## Methods

We collected cerebrospinal fluid samples from patients among hospitals of Turin between 2001 and 2018, undergoing lumbar puncture (LP) for clinical reasons. All of them were morning LP. Patients signed a written informed consent for CSF withdrawal, storage and analysis. The retrospective analysis of the collected data was approved by the Ethics Committee (Città della Salute e della Scienza, Ospedale Molinette, RetroNEG Protocol, n 0094995, October 4th 2017). Inclusion criteria comprised patients with confirmed *Mycobacterium tuberculosis* meningitis (positive *M. tuberculosis* DNA or culture on CAF). All cases were microbiologically confirmed TBM (either with CSF PCR or culture) but CT values were not available. AD and control participants were included from an ongoing study on CSF and nasal brushing biomarkers (“SOLFAMU” study, NCT02951559). AD participants had a confirmed diagnosis of AD (by a combination of cognitive performance, clinical history, genetics and imaging studies). Control participants were those with syphilis that underwent LPS for excluding CNS involvement with a negative CSF Veneral Disease Research Laboratory (VDRL). We studied biomarkers of blood-brain-barrier (BBB) permeability (CSF to serum albumin ratio, “CSAR”), inflammation (CSF to serum IgG ratio, neopterin), amyloid deposition (Aβ1–42), neuronal damage [Total tau (T-tau), Phosphorylated tau (P-tau), 14-3-3 protein) and astrocyte damage (S-100 β) [1–3, 19]. Quantitative determination of albumin in serum and CSF was measured by Immunoturbidimetric methods (AU 5800. Beckman Coulter, Brea, CA. USA), 14-3-3 protein was measured by immunoenzymatic methods (ELISA) (Santa Cruz Biotechnology); CSF tau, P-tau and Aβ1–42 were measured by immunoenzymatic methods (Fujirebio diagnostics, Malvern. U.S.A.). Neopterin and S-100β were measured through validated ELISA methods [DRG Diagnostics (Marburg, Germany) and DIAMETRA S.r.l. (Spello, Italy), respectively]. Reference values were as follows: CSAR [< 6.5 (up to 35 years) and < 8.0 if aged above 35 years], IgG ratio (< 0.7), 14.3.3 protein (normally absent), T-tau [< 300 pg/mL (patients aged 21–50), < 450 pg/mL (patients aged 51–70) or < 500 pg/mL in older patients], P-tau (< 61 pg/mL), Aβ1–42 (> 770 pg/mL), neopterin (< 1.5 ng/mL) and S-100β (< 380 pg/mL) [1–3, 19]. Imaging (either MRI or CT) and eletrophysiological studies (EEG) were performed for all patients. Data were analyzed using non-parametric tests: variables were described as number (percentage) with medians [interquartile ranges (IQR)]. Additionally, we used Spearman’s test for bivariate analysis and Mann-Whitney’s/Kruskal-Wallis’s tests for group comparisons. Data analysis was performed using SPSS software for Mac (version 26.0. IBM Corp). Graphs were created with both SPSS and PRISMA.

## Results

**Fig. 1:**
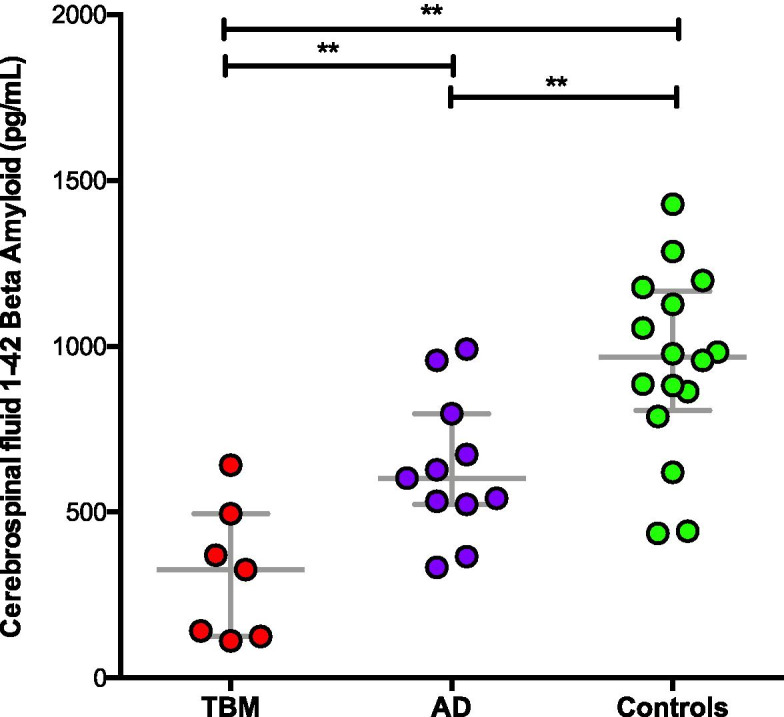
Levels of TBM 1–42 Beta Amyloid compared to AD and control groups. (p = < 0.001)

**Fig. 2:**
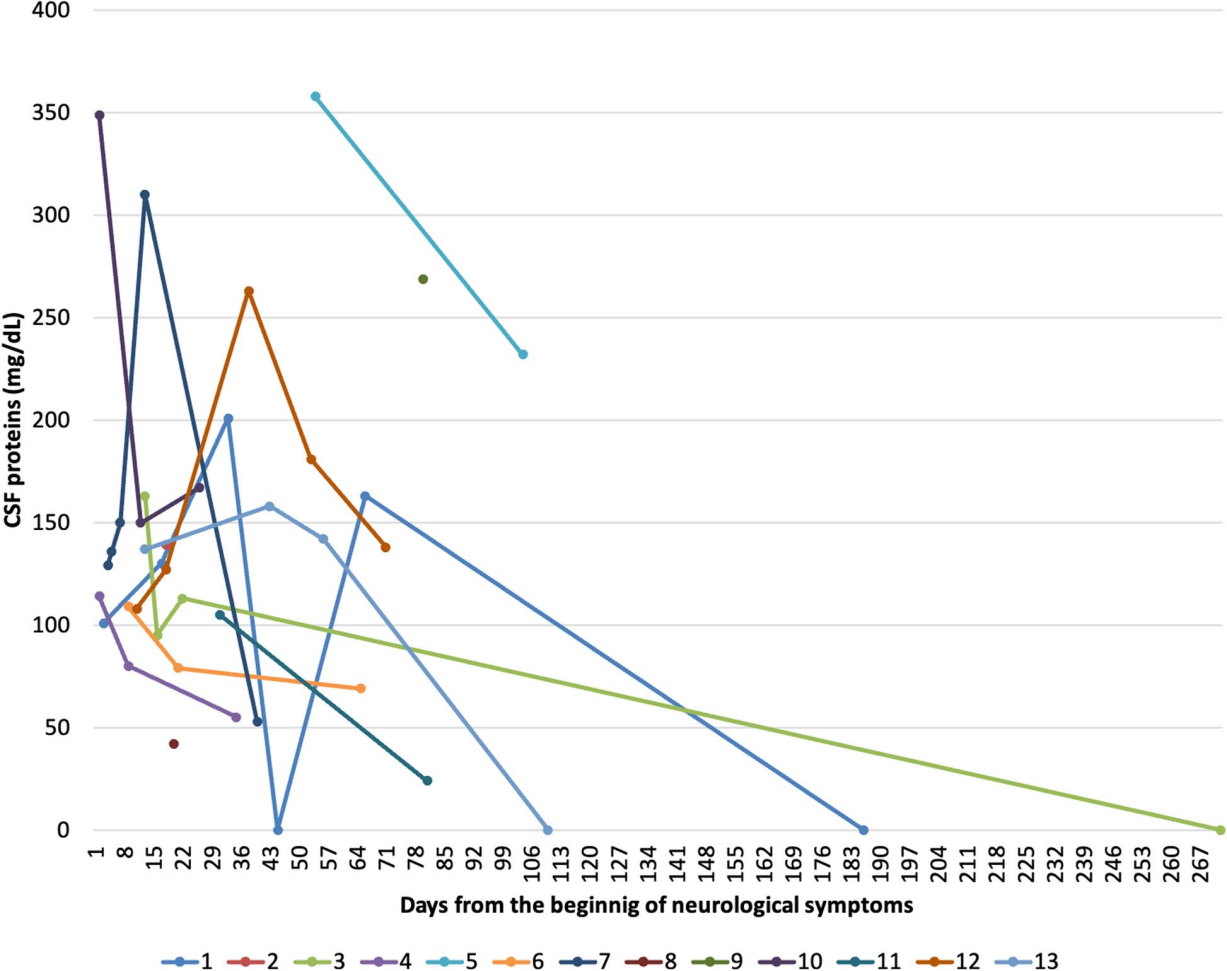
Variation over time for CSF protein. The red line indicates Normal values. Data in mg/dl

**Fig. 3:**
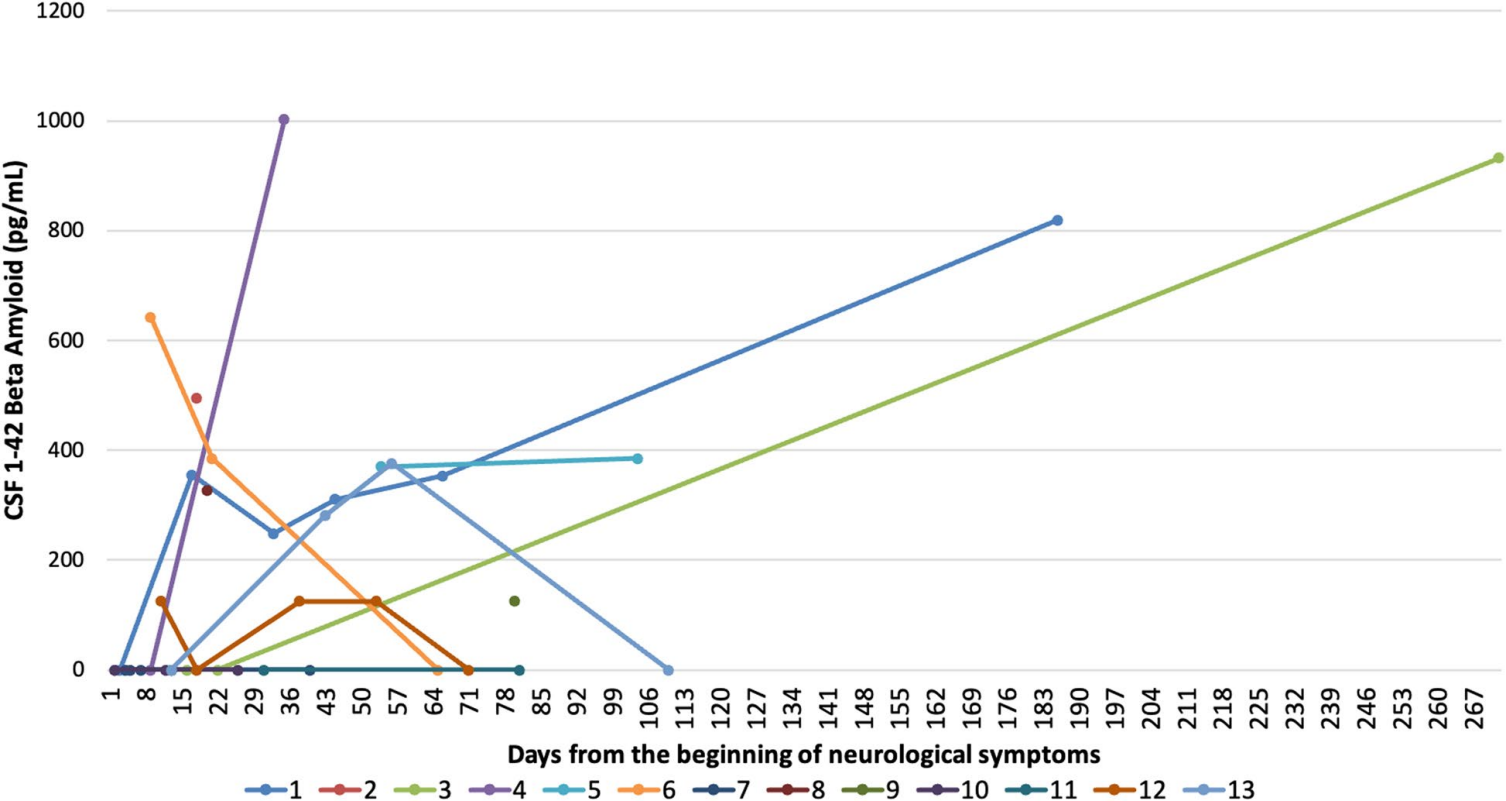
Variation over time for amyloid-beta. The red line indicates Normal Values (> 770) Data in pg/ml

**Fig. 4:**
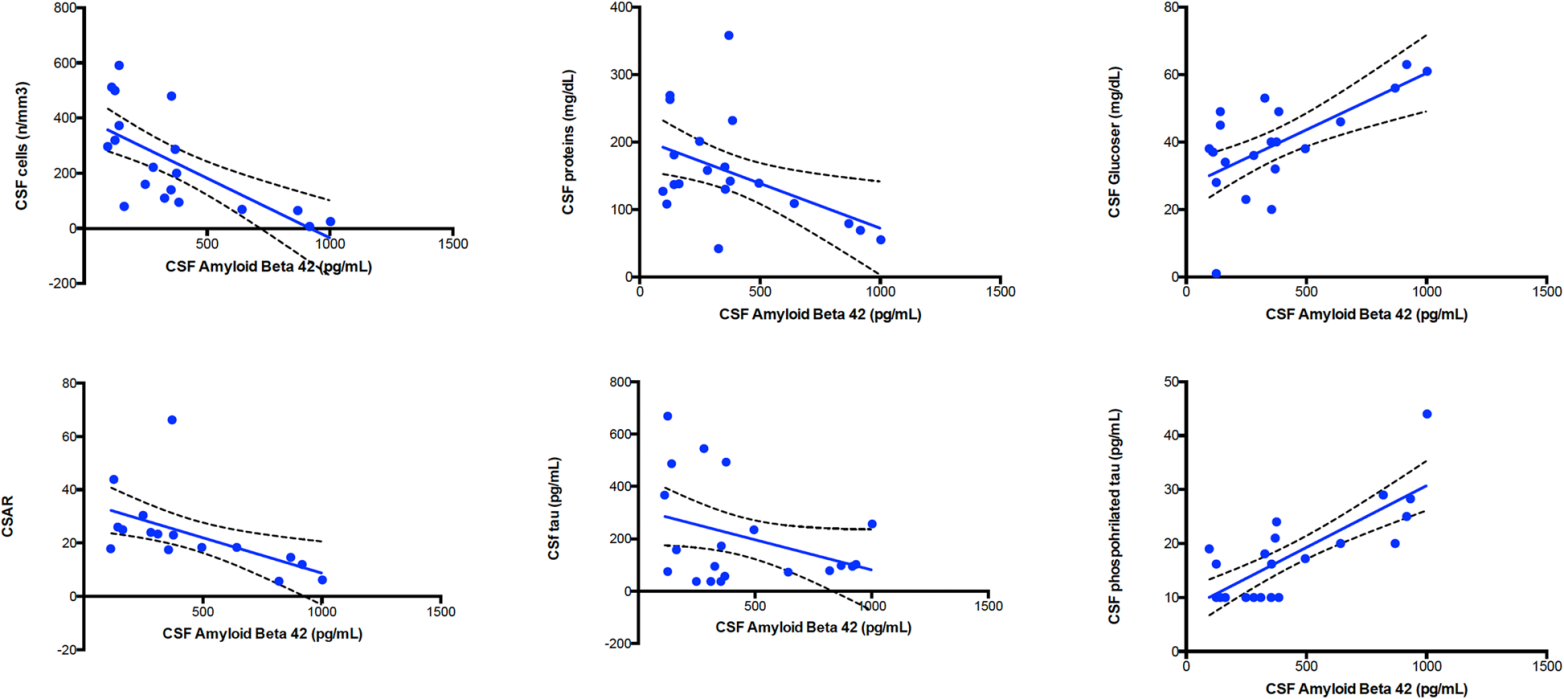
Grouped biomarkers analyzed trough spearman test for bivariate. CSF 1–42 Amyloid-Beta (x) correlates with classical TBM findings and altered neuromarkers: CSF cells (rho= -0.777, p = 0.009), CSF glucose (rho 0.568, p = 0.009), CSAR (rho − 0.690 p = 0.004), P-Tau (rho 0.717, p = 0.04)

Thirteen TBM patients were included: 10 (83 %) were male, 8 (67 %) Caucasian, median age was 51 [IQR 24.5–63.5]. All tested negative for HIV and viral hepatitis, and no other reasons for immunosuppression were found. In TBM group, two participants (15 %) showed hypertension as comorbidity, 2 (15 %) diabetes and 1 (7 %) hypothyroidism, none renal impairment; 7 (58.3 %) showed focal or diffuse imaging abnormalities at CT/MRI scans and 2/13 (15 %) had EEG alterations. Baseline CSF parameters showed typical TBM findings: 150 CSF cells/mm3 [IQR 50–245], 129 mg/dL of proteins [IQR 5-109] and 32 mg/dL of glucose [IQR 25.5–45.5]. Median age was 68.5 (62–76 IQR) for AD and 48 (40–56 IQR) for controls, respectively. Sex resulted 6/11 (55 %) male for AD and 16/16 (100 %) for controls. CSF biomarkers are described in Tables 1 and 2. Values outside ranges were observed for CSAR [18.4 (IQR 17.1–30.9)], neopterin [14.3 ng/ml (IQR 9.8–18.8)], IgG ratios [15.4 (IQR 7.9–24.9)] and 14.3.3 (positive, 5/13, 38,5 %); very low levels of CSF Aβ1–42 were observed [348.5 pg/mL (IQR 125-532.2)], lower than values in the control-groups, [603 pg/mL (IQR 528–797) and 978 (IQR 789–1178), AD and controls respectively], Fig. 1. CSF proteins, P-Tau and Total-Tau between groups were consistent with classical CSF own group’s findings, CSF proteins and P-Tau being significantly higher and lower, respectively, in TBM group (Supplementary Fig. 1). Seven days [1-7.5] lasted from symptoms’ onset to first LP, 13 days [IQR 4-32] to second LP and 7.5 days [1-14] to treatment. 10 patients received more than one LP with a median of 3 [2–4]: time course of CSF proteins and Aβ1–42 is shown in Figs. 2 and 3. We also performed tests for grouped patients choosing seven days intervals for clinical characteristics, Supplementary Fig. 2. We analyzed all available CSF biomarkers per patient and calculated the correlation among them (at the same time point for a total of 66 pairs): CSF Aβ1–42 was associated with CSF cells (rho= -0.777, p = 0.009), CSF glucose (rho = 0.568, p = 0.009), CSAR (rho − 0.690 p = 0.004), P-Tau (rho = 0.717, p = 0.04), Fig. 4. All patients were treated with standard TBM regimens; 5 (38 %) and 2 (15 %) received additional fluoroquinolones or linezolid. All patients received high dose steroid as adjunctive therapy. One patient had disseminated tuberculosis. No concomitant infections were recorded. No patient died; 7 subjects (54 %) survived but suffer long-term disability and 6 (46 %) survived with no consequences. We exploratory observed a non-statistically significant difference between CSF Aβ1–42 (at admission in our ward, second LP received by patients) in those who suffered sequelae versus those who did not [142 vs. 568 pg/mL, p = 0.095) (Supplementary Fig. 3).

**Table 1:** Neuromarkers, median values in TBM group

	Median	Percentile 25	Percentile 75
**42BetaAmyloid** **(pg/ml)**	348.6	125	532.2
**P-tau** **(pg/ml)**	18.1	16.7	20.5
**T-tau** **(pg/ml)**	85.1	61	333.9
**IgG ratio**	15.4	7.9	24.9
**CSAR**	18.4	17.1	30.9
**CSF glucose** **(mg/dl)**	32	25.5	42.5
**CSF proteins** **(mg/dl)**	129	106.5	216
**CSF Cells** **(n/mm3)**	150	84.5	330
**Delta symptoms** **(Days)**	7	1	7.5

**Table 2:** Population features

Percentage of values outside reference range (Baseline)	TBM patients
**14.3.3.**	38.5 %
**Neopterin**	15.5 %
**T-tau**	0 %
**P-tau**	15.5 %
**42 BetaAmyloid**	100 %
**CSAR**	100 %
**IgG Ratio**	100 %
**CSF Proteines**	93 %
**CSF Glucose**	93 %
**S100Beta**	0 %
**Days from symptoms to treatment**	7.5 (IQR 1-14)
**Abnormal brain MRI**	53 (%)
Diffused	38.5 (%)
Focal	61.5 (%)
**Abnormal EEG**	15 (%)

## Discussion

In this small case series study we measured several cerebrospinal fluid biomarkers in meningeal tuberculosis. Obtaining morning LP was of added value because standardised specimen sampling. We confirmed the presence of classical TBM CSF findings such as BBB impairment, inflammation and report here, for the first time, very low level of Aβ1–42 [1–3, 19, 20], even lower than what we measured in AD control group (Fig. 1). Hyperphosphorylated tau CSF concentrations were very low in TBM even when compared to controls: this may suggest an inflammatory impairment of tau metabolism that needs to be confirmed in patients’ follow up (Supplementary Fig. 1). Neuronal damage is a classical feature of TBM due to its devastating inflammation and disruptive process. 14.3.3 positivity was found in 5/13 (38,5 %) of TBM; this cellular-cycle protein, previously associated with prionic disease, accumulates in the CSF after neuronal damage especially during bacterial involvement of CNS and it is cleared from the CSF after successful treatment [21]. BBB impairment and IgG synthesis were observed; CSAR and IgG ratios were high in TBM, confirming results in literature where a significant impairment in BBB due to TBM is described [3, 20]. A raised level of neopterin can be found in TBM, denoting intrathecal production by macrophage-derived cells and, as the BBB has a low permeability for peripheral neopterin, it represents a relevant index of local inflammation. [9, 21, 22] Moreover, we found out that classical markers of TBM disease activity had a good correlation with Aβ1–42: low glucose and higher cells correlates with lower amyloid, BBB damage expressed by CSAR, as well as P-Tau, resulted higher in lower Aβ1–42 (Fig. 4) [20, 23]. These findings outline the possibility for amyloid-beta of being a good proxy of precocious disease activity and a potential marker to follow over time. Also, lower Aβ1–42 level was associated with worse outcomes, thus suggesting a possible prognostic of this marker in clinical practice (**Supplementary Fig. 3**). Additionally, the observation of low levels of Aβ1–42 in patients with TBM is of potential interest and should be interpreted in the context of the recent discovery of a possible antimicrobial role of amyloid-beta [15, 24–26] and of a hypothetical infectious “trigger” for AD [27]. Amyloid-beta protein seems to be shed and playing an anti-infective role in response of several infections in a murine model [28]. In vivo low levels of CSF amyloid-beta have been observed in patients with pneumococcal meningitis and other bacterial meningitis [19, 29] That is critical because observing amyloid metabolic alterations during TBM is perhaps the key passage for understanding amyloid’s antimicrobial role. This may show how amyloid metabolism is potentially altered by several infections, as seen for HSV6 and 7 that have been recently associated with development of AD, probably playing an important role in driving alterations such oxidative damage and progression to accumulation of neurofibrillary tangles. In addition to these reports, the most abundant data for this association are available for HSV-1 encephalitis. In this setting the link with AD (characterized by amyloid deposition and tau pathology) has been repeatedly suggested [7, 10, 11, 18, 30–33]. Long-time implications for the lower level of Aβ1–42 are under discussion but may be in the future linked to cognitive function assessed by serial neuropsychological tests. Yet it should be acknowledged that amyloid deposition increases with age: despite not being, alone, diagnostic for AD it has been associated with poorer cognitive performance in non demented adults [34]. To remain in the field of neurodegenerative diseases, nationwide studies associated a previous diagnosis of TB with Parkinson’s disease although confirmatory studies are currently lacking [35]; putting together these data, it is noteworthy the finding that associated longitudinally collected plasma Aβ1–42 concentrations with cognitive decline in patients with mild cognitive impairment [36, 37]. Several mechanisms regarding the finding of low Aβ1–42, besides amyloid deposition in the brain parenchyma, can be hypothesized. Amyloid-beta levels could be reduced because of the interaction of amyloid-beta fragments with albumin, usually elevated in CSF TBM, thus lowering levels of the free peptide. Additionally, Aβ1–42 can cross the BBB by leaking in CNS and then accumulating (even if it is known that in peripheral tissues Aβ1–40 is prevalent), in the context of increased permeability, thus being lower in the CSF/CNS. Data on the potential measurement of serum amyloid-beta peptides in the setting of AD may confirm this hypothesis [20, 22, 38]. Another mechanism could be an impaired and reduced amyloid-beta clearance from the CNS [29]: the ISF/CSF flow is believed now to be partially convective and through perivascular spaces that can be harmed during tubercular infections of the CNS and systemic inflammation [8]. That could be particularly relevant following the recent discovery of the so called Glymphatic Central Nervous System [39, 40]. TBM it is known to affect the basal anatomic section of the brain with a reduced CSF recirculation, a fibrosing effect and a possible central hypertensive syndrome. In view of these observations it is possible that even the glymphatic recirculation is altered; unfortunately, data are scarce and there are no reliable markers up to date.

To conclude, the analysis regarding the time to normalization for Aβ1–42 in our population deserves an additional remark: relying on our data, only three patients normalized amyloid-beta during follow-up. Patient 4 at day 22, patient 1 at day 190, patient 3 at day 267 (Fig. 3). Acknowledging that data are limited and we were not able to measure these equally for all patients, it is still of great interest that the vast majority of patients did not normalize amyloid-beta while hospitalized nor under treatment; moreover, the time to normalization was not homogenous between patients suggesting a persistent and unpredictable ongoing accumulation and probable undergoing slight but constant and enduring inflammation, which is coherent with TBM physiopathology and such a life-threatening condition. Following a recent article and debate [4, 5], amyloid-beta could be tested as a prognostic marker in both pediatric and adult population, backing the routine use of available neuromarkers for both a better tailored approach to patients and in research. An adjunctive information may come from retesting Aβ1–42 levels at the end of therapy (one-year follow-up retesting). To our knowledge, this is the first case showing such low levels of Aβ1–42 in TBM; its accumulation, drove by neuroinflammation related to infections, can be central in understanding neurodegenerative diseases. This study has several limitations: sample size, impossibility to perform homogenous number of LP at follow-up for all patients, and incomplete data on neurofilaments (NFL). Also, analyzing grouped TBM markers over time we could not highlight any particular pattern, probably due to the variety of performing LP at different moments of the disease (Supplementary Fig. 2). An additional limitation pertains to the smaller number of patients for the outcome comparison analysis (sequelae vs. non-sequelae, Supplementary Fig. 3). Ultimately, in this small case series we had no deceased participant; we were able to present exploratory results suggesting that those reaching the lowest CSF Aβ1–42 levels have neurological sequelae. We were not able to infer, from our data, if this was due to a longer disease course, poor antimycobacterial penetration/efficacy or other (including genetic) factors. Nevertheless, the finding of low Aβ1–42 concentrations, confirmed when compared to control-groups, and even lower than what was measured in AD, and its potential relation with others TBM indicators, both clinical and laboratory, warrant further analysis in controlled and larger settings. Further studies may also aim at characterizing patients that lacked Aβ1–42 normalization despite clinical effectiveness, but also the long-term neuropsychological outcome of TBM survivors.

## Conclusions

CSF Biomarkers from patients with TBM were compatible with inflammation, blood brain barrier damage and impairment in beta amyloid metabolism. Aβ1–42 could be tested as a prognostic marker, backing the routine use of available neuromarkers for both a better tailored approach to patients and in research. To our knowledge, this is the first case showing such low levels of Aβ1–42 in TBM; its accumulation, drove by neuroinflammation related to infections, can be central in understanding neurodegenerative diseases. Further studies are needed in order to understand the relevance of these observations.

## Supplementary Information

**Additional file 1.**

**Additional file 2.**

**Additional file 3.**

## Data Availability

The data that support the findings of this study are available from “Città della Salute e della Scienza, Ospedale Molinette, RetroNEG Protocol, n 0094995, October 4th 2017”; “SOLFAMU” study, NCT02951559. The datasets used and/or analysed during the current study are available from the corresponding author on reasonable request.

## Abbreviations

TBM: Tuberculous meningitis
NSE: neuron-specific enolase
AD: Alzheimer Disease
CNS: Central nervous system
LP: lumbar puncture
BBB: Blood-brain barrier
CSF: cerebrospinal fluid
HHV6: Human Herpes Virus 6
Aβ1-42: 1-42 Amyloid-βeta
