# Supplementary material for: Low cerebrospinal fluid Amyloid-βeta 1–42 in patients with tuberculous meningitis
Source: BMC Neurol. 2021 Nov 16;21:449. doi: 10.1186/s12883-021-02468-2 (PMC8594191; doi:10.1186/s12883-021-02468-2)
Supplement: Supplementary file 1 — Additional file 1. [file 12883_2021_2468_MOESM1_ESM.docx]

**Supplementary figure 1.** Levels of TBM CSF proteins, P-Tau and Tau compared to AD and healthy control groups. CSF proteins (p=<0.0001),P-Tau (p=<0.0001) and Tau (p=0.07).
