# Supplementary material for: Low cerebrospinal fluid Amyloid-βeta 1–42 in patients with tuberculous meningitis
Source: BMC Neurol. 2021 Nov 16;21:449. doi: 10.1186/s12883-021-02468-2 (PMC8594191; doi:10.1186/s12883-021-02468-2)
Supplement: Supplementary file 2 — Additional file 2. [file 12883_2021_2468_MOESM2_ESM.docx]

**Supplementary figure 2.** Changes of CSF proteins and Aβ1-42 in TBM group over time (grouped median values at baseline and at 7 days interval, repeated).
