# Supplementary figures and images for: Low cerebrospinal fluid Amyloid-βeta 1–42 in patients with tuberculous meningitis
Source: BMC Neurol. 2021 Nov 16;21:449. doi: 10.1186/s12883-021-02468-2 (PMC8594191; doi:10.1186/s12883-021-02468-2)

**
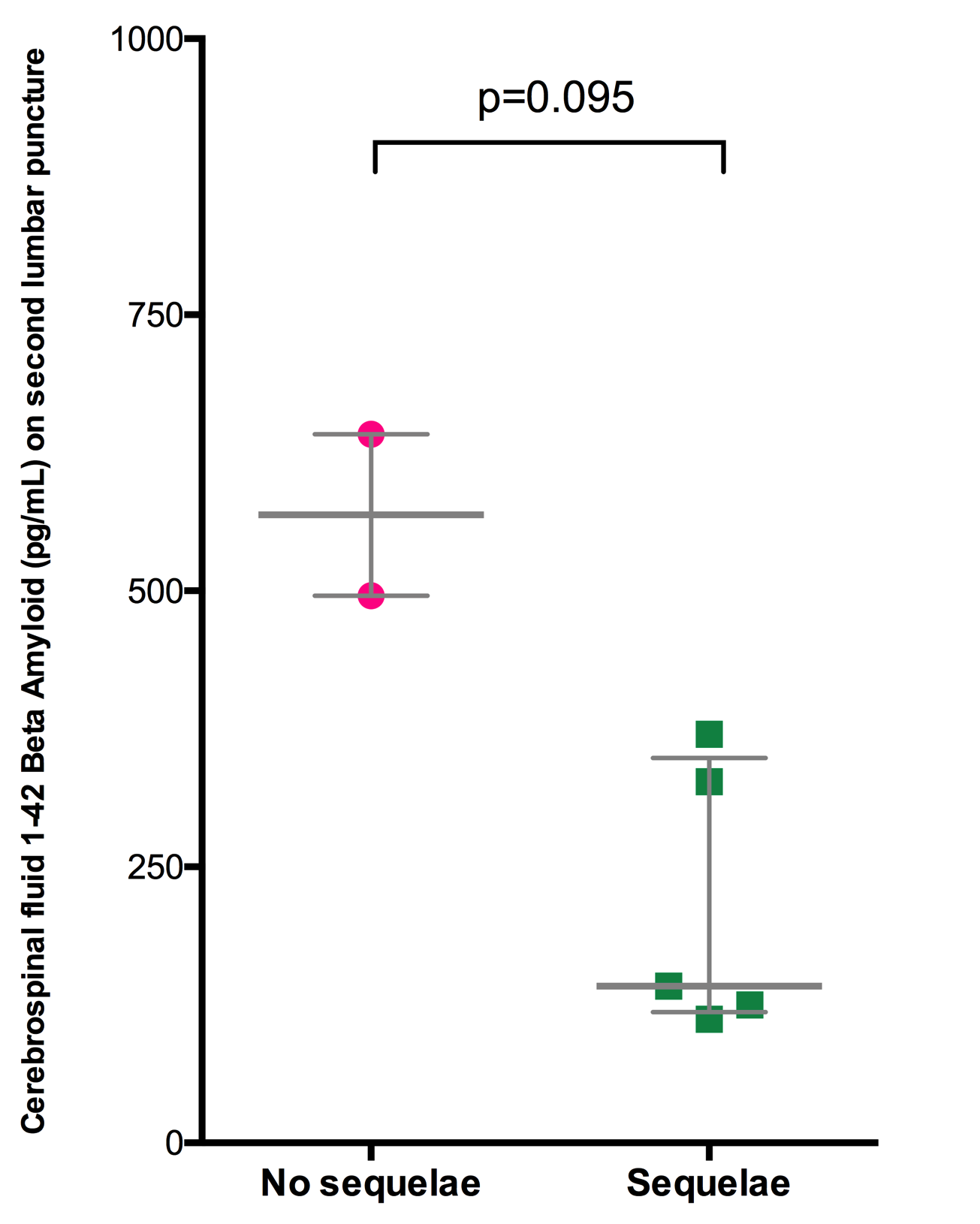
**

**Supplementary figure 3:** Beta Amyloid 1-42 and correlation with outcome (p = 0.095)

Supplement: Supplementary file 3 — Additional file 3. [file 12883_2021_2468_MOESM3_ESM.docx]
